# Long Noncoding RNA MALAT1: Salt-Sensitive Hypertension

**DOI:** 10.3390/ijms25105507

**Published:** 2024-05-18

**Authors:** Mohd Mabood Khan, Annet Kirabo

**Affiliations:** Department of Medicine, Preston Research Building, Vanderbilt University Medical Centre, Nashville, TN 37232, USA

**Keywords:** long noncoding RNA, MALAT1, salt, hypertension, inflammation

## Abstract

Hypertension stands as the leading global cause of mortality, affecting one billion individuals and serving as a crucial risk indicator for cardiovascular morbidity and mortality. Elevated salt intake triggers inflammation and hypertension by activating antigen-presenting cells (APCs). We found that one of the primary reasons behind this pro-inflammatory response is the epithelial sodium channel (ENaC), responsible for transporting sodium ions into APCs and the activation of NADPH oxidase, leading to increased oxidative stress. Oxidative stress increases lipid peroxidation and the formation of pro-inflammatory isolevuglandins (IsoLG). Long noncoding RNAs (lncRNAs) play a crucial role in regulating gene expression, and MALAT1, broadly expressed across cell types, including blood vessels and inflammatory cells, is also associated with inflammation regulation. In hypertension, the decreased transcriptional activity of nuclear factor erythroid 2-related factor 2 (Nrf2 or Nfe2l2) correlates with heightened oxidative stress in APCs and impaired control of various antioxidant genes. Kelch-like ECH-associated protein 1 (Keap1), an intracellular inhibitor of Nrf2, exhibits elevated levels of hypertension. Sodium, through an increase in Sp1 transcription factor binding at its promoter, upregulates MALAT1 expression. Silencing MALAT1 inhibits sodium-induced *Keap1* upregulation, facilitating the nuclear translocation of Nrf2 and subsequent antioxidant gene transcription. Thus, MALAT1, acting via the Keap1-Nrf2 pathway, modulates antioxidant defense in hypertension. This review explores the potential role of the lncRNA MALAT1 in controlling the Keap1-Nrf2-antioxidant defense pathway in salt-induced hypertension. The inhibition of MALAT1 holds therapeutic potential for the progression of salt-induced hypertension and cardiovascular disease (CVD).

## 1. Introduction

Hypertension, a major contributor to cardiovascular disease (CVD), has reached alarming levels. It has a huge impact on global health, impacting over 1 billion adults globally, and its incidence is steadily increasing, adding significantly to the worldwide disease burden [1,2]. Underlining the significance of early identification and intervention, the American College of Cardiology (ACC) and the American Heart Association (AHA) revised their high blood pressure (BP) guidelines in 2017, lowering the threshold from 140/90 mmHg to 130/80 mmHg [3]. The development of hypertension involves a complex interplay of genetic and environmental factors, encompassing critical elements such as the renin–angiotensin–aldosterone system, thrombogenesis, decreased platelet function, and the sympathetic nervous system [4,5,6,7,8]. Therapeutic drug design has concentrated on genes and their associated proteins within these signaling pathways. Despite the effectiveness demonstrated by various drug classes in reducing cardiovascular mortality (by 33%), major adverse cardiovascular events (by 29%), and heart failure (by 37%), hypertension remains a significant global public health challenge [9]. As a result, there is an urgent need to deepen our understanding of the underlying mechanisms of hypertension and develop more precise and targeted therapeutic approaches [10].

Salt sensitivity is a characteristic that causes BP variations according to salt intake, affecting approximately 50% of those with hypertension and 25% of those without hypertension [11]. The intake of sodium (Na^+^) should not exceed 2300 milligrams per day, as recommended by the AHA. Our earlier studies with mouse models showed that high salt levels activate Antigen Presenting Cells (APCs), including monocytes and dendritic cells (DCs), via the production of immunogenic isolevuglandins (IsoLG), leading to high BP [12,13]. We reported that salt-sensing kinase serum/glucocorticoid kinase 1 (*SGK1*) in APCs increases salt-sensitive hypertension through activating the NADPH oxidase, and it also facilitates the salt-induced production and assembly of epithelial sodium channels (ENaCα and ENaCγ) [14]. We subsequently found that human APCs express a unique ENaC channel composed of the delta subunit which plays a role in salt-sensitive hypertension via IsoLG production [15]. Additionally, we have discovered a novel mechanism by which the activation of the *NLRP3* inflammasome in APCs contributes to salt-sensitive hypertension through the ENaC and production of IsoLG-adducts [16]. In additional studies, we found that high salt consumption induces dysbiosis, which leads to high BP through the production of IsoLG adducts in myeloid APCs [17]. These studies indicate that immune cell activation via ENaC plays a role in salt-sensitive hypertension, but the mechanisms are not fully understood.

Long noncoding RNAs (lncRNAs), exceeding 200 nucleotides, and microRNAs (miRNAs), typically around 21 nucleotides, represent two distinct types of noncoding RNAs (ncRNAs) devoid of protein-coding capacity [18,19]. These ncRNA variants play pivotal roles in governing gene transcription and translation by interacting with the 3’ untranslated region (3’UTR) of messenger RNA (mRNA) [20,21,22]. Functionally, lncRNAs and miRNAs are instrumental in modulating a wide array of cellular processes, including cell proliferation, differentiation, and apoptosis [23,24,25]. LncRNAs also regulate gene expression via mechanisms including competitive binding to miRNAs. For example, according to recent research, lncRNA PCED1B-AS1 directly binds to *miR-3681-3p*, which targets *MAP2K7*, acting as a sponge [26]. A notable example of an lncRNA is metastasis-associated lung adenocarcinoma transcript 1 (MALAT1), also known as nuclear-enriched abundant transcript 2 (NEAT2), initially identified in nonsmall cell lung cancer (NSCLC) [27]. The gene encoding MALAT1 is situated on chromosome 19qA in mice and chromosome 11q13.1 in humans [28]. In humans, the primary MALAT1 transcript is approximately 8 kb long, while in mice, it spans 6.7 kb and lacks intron and Poly A tail [29]. Rather, the MALAT1 transcript goes through a unique 3′ end-processing step wherein RNase P cleaves it to produce a short RNA (mascRNA) that resembles tRNA and is then delivered to the cytoplasm. The cleaved transcript’s 5′ main section develops a triple-helical structure at its 3′ end to prevent degradation [30]. However, studies showed that MALAT1 exclusively functions as a nuclear long noncoding RNA. However, studies have also found MALAT1 in the cytoplasm of several cancer cell types, such as bladder, hepatic, and breast cancer, as well as in the cytoplasm of platelet precursor cells [31,32,33,34].

MALAT1 plays a crucial role in controlling atherosclerosis by modulating the number and activities of inflammatory cells [35]. MALAT1’s influence extends across various pathophysiological processes, including tissue inflammation, embryo implantation, angiogenesis, cardiovascular remodeling, and tumor progression [36]. Elevated levels of MALAT1 expression in hypertension imply its potential abnormal expression in this disease [37]. Furthermore, MALAT1 dysregulation has been linked to numerous human disorders, including gastric cancer [38] and pulmonary arterial hypertension [39]. MALAT1 also works as a competitive endogenous RNA (ceRNA) by sponging miRNAs, which lowers the activity of miRNAs and stops them from interacting with their target genes [40]. The study showed that the lncRNA MALAT1 could bind to miR-145-5p well. They found that compared to wistar kyoto rats, spontaneously hypertensive rats have a downregulated expression of miRNA-145-5p in their thoracic aortas [41]. Furthermore, numerous studies have extensively acknowledged the roles of miR-145-5p in tumor cell migration and proliferation, as well as pulmonary hypertension [42,43]. According to the latest study, MALAT1 acts like a sponge for miR-217-5p, which causes the expression of *HIF-1α* to rise [44]. Another recent study (2024) demonstrated that MALAT1 acts as an endogenous sponge to directly attach to miR-30b-5p and lower its effective expression, enhancing *ATG5* expression and endothelial autophagy [45]. Furthermore, MALAT1 competed with miR-211-5p to raise *FOXO3* levels and repair ovarian function as well [46].

MALAT1 has been identified as a regulator of splicing and epigenetic gene regulation; it was initially linked to the metastasis of lung tumors [27]. MALAT1 interacts with polycomb 2 (CBX4) to moderate histone changes and influence cellular growth [47]. In addition, MALAT1 was found to regulate splicing in HeLa cells by interacting with proteins rich in serine and arginine, which leads to modulation of the subcellular localization of splicing factors [48]. Research reported that rats and hypertensive patients have upregulated the expression of the lncRNA MALAT1 while inhibiting it effectively reduces blood pressure [37]. Furthermore, lncRNA MALAT1 enhances ventricular remodeling in hypertensive rats by blocking MyoD [49]. The lncRNA MALAT1 modulates miR-150-5p/ET1 to control pregnancy-induced hypertension [50]. Another study finds that lncRNA MALAT1 controls the growth, movement, and phenotypic change of vascular smooth muscle cells (VSMCs) through the miR-145-5p/*HK2* axis in hypertension [51]. They reported that the knockdown of lncRNA MALAT1 reversed the Ang II-induced phenotypic transition of VSMCs (reduction of a-SMA, TAGLN, and the overexpression of cyclin D1, PCNA). Previous investigations have revealed that MALAT1 can act as a prognostic or diagnostic marker for various human disorders [52], and accumulating evidence suggests that targeting MALAT1 could represent a potential therapeutic strategy [53] for salt-sensitive hypertension.

In this review, we have compiled a body of evidence to examine the impact of MALAT1 on hypertension and CVD, elucidating its roles in inflammation responses and mechanistic approach via the Keap1-Nrf2 pathway modulates antioxidant defense in salt-sensitive hypertension. Furthermore, we explore targeted therapeutic approaches for the treatment of hypertension.

## 2. Synthesis of Long Noncoding RNA: MALAT1

There are two broad categories of RNA-producing genes: those that code for proteins and those that do not. Approximately 20,000 of the 180,000 transcripts found in human cells are responsible for the coding of proteins, while the remainder of 160,000 transcripts do not code for proteins [54]. Depending on their length, ncRNAs are classified as either small (<200 nucleotides), like miRNAs/miRs, or long (>200 nucleotides), like lncRNAs [55]. Current data suggest that there are more than 100,000 lncRNAs [56,57]. Some features of the biogenesis of mRNA and lncRNA are similar despite there being distinctions between the two [58,59]. To be synthesised, lncRNAs need a template derived from one of the two strands of DNA, much like mRNAs need. Most lncRNAs have m7 G cap at their 5’ ends and poly adenylation at their 3’ ends, and they are transcribed by RNA polymerase II (pol II) [60]. IncRNAs differ from mRNAs in several ways: they are not as well conserved in evolution, they have fewer exons and are expressed at lower levels, and a major fraction of IncRNAs is kept in the nucleus [61].

A growing body of evidence suggests that lncRNAs are critical regulators of gene expression processes like gene suppression and gene activation. In addition, lncRNA regulation can influence mRNA transcription, splicing, translation, output, import, and stability [62]. We will investigate the function of lncRNA MALATs in salt-sensitive hypertension in-depth in the following section.

## 3. Molecular Functions of Long Noncoding RNA: MALAT1

Most lncRNAs are found in the nucleus, but some are also found in the cytoplasm, just like protein-coding RNAs. This subcellular localization is critical to the lncRNAs’ roles. Nuclear lncRNAs primarily exert their regulatory influence on transcription by directing chromatin modifiers [63], and cytoplasmic lncRNAs regulate mRNA translation or affect protein trafficking in the cytoplasm. LncRNAs have been broadly categorised into signals, decoys, guides, and scaffolds based on classic examples of molecular roles. The signal lncRNAs have the potential to spatially and time-dependently regulate gene expression, decoy lncRNAs can either manipulate miRNAs away from their targets or divert transcription factors away from chromatin, guide lncRNAs act as molecular chaperones and can bring chromatin-modifying enzymes to genes in close proximity (in cis) or far away (in trans), and scaffold lncRNAs facilitate histone modification by collecting proteins to create ribonucleoprotein complexes [64,65,66]. 

Various researchers have described the molecular functions of MALAT1, including its role in controlling alternative splicing. Nuclear-retained MALAT1 regulates alternative splicing by modulating the phosphorylation of serine- and arginine-rich (SR) splicing factors [48]. Furthermore, Zong et al. provided more evidence that MALAT1 is important for RNA processing by elucidating its function in RNA splicing [67]. Further work conducted by Bernard et al. sheds light on the function of MALAT1 in normal cellular physiology, namely its function in synaptogenesis through the regulation of specific gene transcription [68].

## 4. Role of Long Noncoding RNA in Salt-Sensitive Hypertension

Despite their potential, lncRNAs remain largely untapped in hypertension research. However, emerging studies indicate that lncRNAs could be crucial in the heart and vascular system’s anatomy, physiology, and pathophysiology [69,70]. One pivotal finding is the role of lncRNAs in modulating the effects of angiotensin II, a key regulator of arterial BP, altering the expression of numerous lncRNAs, including lnc-Ang362, lnc-Ang162, lnc-Ang112, lnc-Ang249, and lnc-Ang219 in VSMCs [71]. Additionally, significant evidence points to the involvement of lncRNAs in salt-sensitive hypertension, elucidating their connection to both salt sensitivity and kidney function [72,73,74]. Gopalakrishnan et al. investigated whether Dahl salt-sensitive and salt-insensitive-13BN rats showed differential expression of lncRNAs [74]. Also, Wang et al. investigated lncRNA differential expression in three different types of rats: Dahl salt-sensitive, salt-insensitive-13BN, and spontaneously hypertensive [75]. In another study, the two most differential lncRNAs were found to be NONRATG007131.2 and NONRATG012674.2 in salt-sensitive hypertension [76]. NONRATG007131.2 has shown a strong association with BP and genes involved in angiogenesis, such as *Matn1*, *Serpinb12*, *Slc39a12*, and *Snap91*, suggesting it may influence BP through vascular development, epithelial cell differentiation, and the transforming growth factor-beta (TGF-beta) signaling pathway. Meanwhile, NONRATG012674.2, which is overexpressed in the high salt sensitivity group, could interact with key hypertension genes like *Anxa8*, *Hspa5*, and *Krt15*, with Hspa5 being part of the HSP70 family associated with hypertension and inflammation [77,78]. This research underscores the significance of NONRATG007131.2 and NONRATG012674.2 in the context of salt-sensitive hypertension.

Reportedly, MALAT1 regulates the activity of endothelial cells (ECs) and the development of new blood vessels [69]. Evidence suggests that SENCR, a lncRNA abundant in vascular cells, contributes to the phenotype of smooth muscle cells [79]. Additionally, it was shown that growth arrest-specific 5 (GAS5) controls the remodeling of caudal, renal, thoracic, and carotid arteries [80]. Additionally, *GAS5* was discovered to be downregulated, which impacted endothelium proliferation and activation in hypertension conditions [80]. Recently, a study examined the impact of goji berries (Lyciumbarbarum L.) on lncRNA in rats fed a high-salt diet. Berries enhanced endothelial nitric oxide synthase (eNOS) expression and reduced sONE lncRNA, which, in turn, alleviated hypertension in borderline hypertensive rats [73].

GenSalt examined single-nucleotide polymorphisms and gene-based interactions with sodium to identify the *SCOC-AS1* gene as a BP locus that interacts with sodium [81]. The lncRNA SLC8A1-AS1 has the potential to control *SLC8A1* [82], which is known to be a salt-sensitive hypertension trigger and highly expressed in vascular smooth muscle [83]. The pathophysiology of salt-sensitive is heavily influenced by endothelial dysfunction, and KCNQ1OT1 may play a pivotal role in mediating these processes [84]. In VSMCs, KCNQ1OT1 interacts with miR-183-3p to increase *CTNNB1* expression, which, in turn, affects VSMC proliferation and death [85]. Furthermore, KCNQ1OT1 has the potential to inhibit intimal hyperplasia-related VSMC inflammation and proliferation [86]. These studies highlight the intricate ways in which lncRNAs can influence BP and offer new avenues for understanding and potentially treating this condition.

## 5. MALAT1 Role in Immune System and Hypertension

Salt-sensitive hypertension exhibits a well-established link with the immune system. The development of hypertension is triggered by elevated salt intake, leading to inflammatory activation and oxidative stress, subsequently causing vascular and renal dysfunction [87]. This complex disease involves various factors, including the innate and adaptive responses of the immune system. Pro-hypertensive stimuli prompt APCs like DCs, macrophages, and T cells to react, activating T cells through antigen-major histocompatibility complex (MHC) receptor interaction and co-stimulation, thereby promoting further immunological inflammation [88]. In co-culture systems where epithelial cells interact with DCs, the upregulation of MALAT1 expression in epithelial cells occurs. Conversely, the downregulation of MALAT1 in this co-culture system results in DCs expressing more mature surface markers (CD80, CD86, and MHC class II), increased production of inflammatory cytokines (TNF-α, IL-6, and IFN-γ), and elevated secretion of chemokines (CXC receptor 2, CXCR4) [89]. The suppression of MALAT1 leads to inhibited cell apoptosis and increased cell viability in DCs co-cultured with epithelial cells, suggesting that MALAT1 regulation influences DCs in ways that alter their development, cytokine production, chemotaxis, and susceptibility to apoptosis [89]. Xue et al. reported that salt hypertensive patients had greater levels of MALAT1 in macrophages (fold change—1.2), and blocking MALAT1 reduced BP in an animal model [37].

Alterations to the artery wall’s ECs, VSMCs, and extracellular matrix have been linked to hypertension [90]. In southern Chinese populations, genetic polymorphisms in MALAT1 have been associated with an increased risk of hypertension development [39]. MALAT1 rs619586 A to G is proposed to directly upregulate the expression of X box-binding protein1 by acting as a competing endogenous RNA for miR-214, thereby limiting the proliferation and migration of vascular ECs in vitro and shortening the S-M phase conversion [39]. Increased MALAT1 expression has also been identified in the pulmonary arteries and pulmonary artery smooth muscle cells (PASMCs) of hypertensive patients [91]. By sponging miR-124-3p.1, which targets Kruppel-like factor 5 (KLF5), MALAT1 enhances HPASMC proliferation and induces G0/G1 cell cycle progression [91]. White-coat hypertension, characterised by increased clinic BP, similarly showed significant upregulation of MALAT1, suggesting its potential use as a biomarker for the diagnosis of hypertension [92]. Research shows that blocking the activity of MALAT1 in cultivated endothelium cells improved sprouting but halted cell cycle progression. Hence, inhibiting MALAT1 could potentially have a therapeutic impact by eliciting an antiangiogenic effect [69].

Brock et al. [93] found that MALAT1 expression was considerably elevated in PASMCs and the lung tissues of animals with hypertension. Furthermore, they proved that MALAT1 controlled the phenotype of PASMCs and enhanced their movement and multiplication. Additionally, the downregulation of MALAT1 was shown to prevent PASMC proliferation and cardiac hypertrophy by increasing the levels of cyclin-dependent kinase inhibitors. Aside from its function in PASMCs, MALAT1 is also essential for pulmonary artery endothelial cells (PAECs). One study found that downregulating MALAT1 boosted mobility and lightly raised the apoptosis of human umbilical vein endothelial cells (HUVECs). The results of a microarray study that aimed to determine MALAT1’s mode of action showed that HUVECs transfected with MALAT1 siRNA showed an upregulation of cell cycle inhibitory genes [69]. Additionally, it has been suggested that MALAT1 is responsible for regulating the characteristic transformation of ECs [94]. Several studies have shown that MALAT1 targets different genes and miRNAs that play a key role in the pathophysiology of hypertension and other disorders whose dysregulation is strongly associated with different pathological outcomes (Table 1).

## 6. Targeted Mechanism of MALAT1- Nrf2/Keap1 in Salt-Sensitive Hypertension

**Figure 1:**
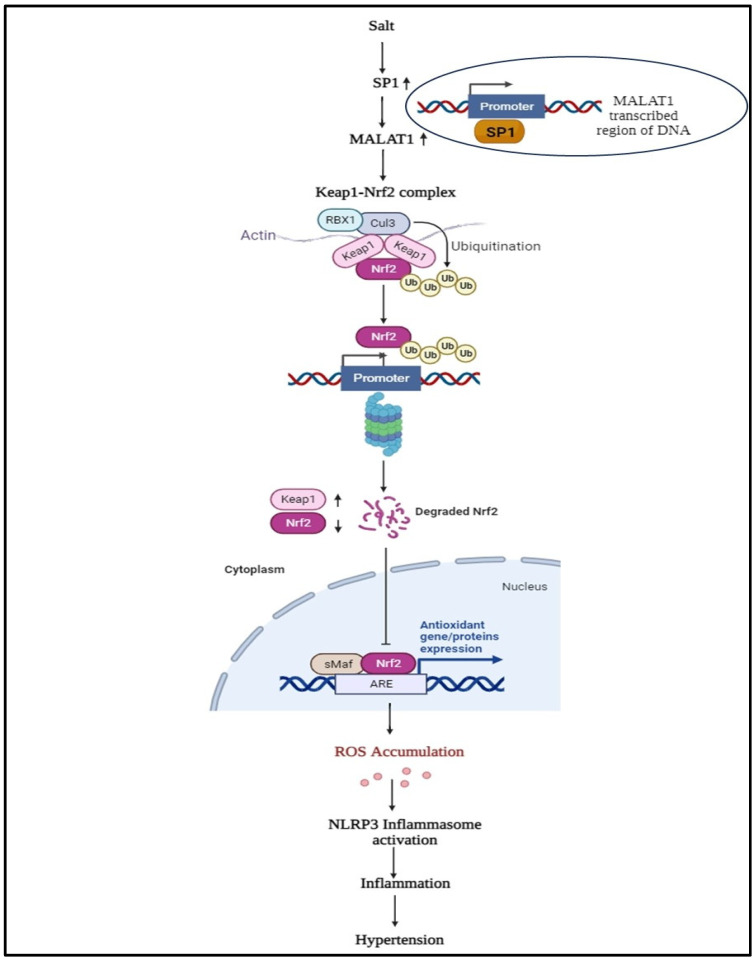
Targeted mechanism of MALAT1- Nrf2/KEAP1 and their role in the development and progression of salt-sensitive hypertension. In high-salt conditions, Sp1 directly binds to MALAT1 and Keap1 (an additional binding site), thereby initiating the cascade. Specifically, MALAT1 promotes Keap1 transcription by enhancing transcription factor binding to the promoter region. When Keap1 levels are high, the master regulator Nrf2 is unable to enter the nucleus and cannot transcribe the genes involved in the antioxidant response. Additionally, Keap1 and Nrf2 are both targets of the ubiquitin ligase Cullin3/Rbx1. Keap1 continuously directs the ubiquitin ligase to degrade Nrf2, which anchors it. This, in turn, leads to the generation of reactive oxygen species (ROS). Accumulated ROS activate the NLRP3 inflammasome, which, in turn, induces the inflammatory response (the production of pro-inflammatory cytokines) that is associated with salt-sensitive hypertension).

The lncRNA MALAT1 plays a crucial role in regulating various pathological conditions, including hypertension, tumors, immunomodulatory disorders, aging, and wound healing, as extensively investigated and documented [37,110,111,112,113]. Furthermore, although lncRNA MALAT1 has been found to play a role in the regulation of inflammation and angiogenesis [114], its role in the maintenance of oxidative stress remains unknown. In this regard, our intriguing data demonstrate that lncRNA MALAT1 also plays a significant role in the maintenance of the antioxidant defense system [115] in salt-sensitive hypertension (Figure 1).

## 7. SP1/MALAT1 Signaling

Specificity protein 1 (Sp1), a widely expressed transcription factor found in various cell types and tissues, features a C-terminal domain with three C2H2-type zinc fingers [116]. These zinc fingers enable Sp1 to recognize and bind to GC-rich regions in DNA [117]. Controlling cell cycle growth and development, Sp1 plays a crucial role as a transcription factor [118]. Research has documented that increased Sp1 expression has been observed in cells associated with various disease types compared to normal cells [119,120]. Research indicates that a high-salt diet elevates myocardial Sp1 levels in the Dahl salt-sensitive hypertensive model [121]. Furthermore, angiotensin causes cardiac fibroblasts to increase Sp1 expression over time [122]. The promoter region of the MALAT1 gene contains two potential binding sites for Sp1 [123]. Therefore, an increase in Sp1 transcription factor binding at the promoter of *MALAT1* has led to an increase in its expression level. To prove this, the reduction of MALAT1 transcription was successfully achieved by silencing the *Sp1* gene and modifying the binding site [123]. To be concise, high salt activates the production of MALAT1, enhancing Sp1 affinity at the *MALAT1* promoter and increasing Sp1 binding at the *Keap1* promoter [115]. The *Keap1* promoter also has a Sp1 binding site (-160/-153) [124], and histone alterations in the *Keap1* promoter enhance Sp1 binding [125]. Keap1 overexpression prevents Nrf2 from entering the nucleus, thereby hindering the transcription of anti-oxidant response enzymes.

## 8. Keap1/Nrf2 Signaling

Keap1/Nrf2 is a signaling pathway that is vital to the cellular antioxidant response. This signaling pathway has been shown to be the most significant endogenous antioxidant pathway in the body because it is able to resist oxidative stress that is induced by both internal and external oxidation as well as chemical stimulation. As a result, it plays a crucial role in the defense against all types of injuries [126]. Various environmental stressors are designed to disrupt normal cellular processes, necessitating adaptive responses governed by well-established regulatory mechanisms. Cellular redox homeostasis, a vital aspect of this adaptation, is orchestrated by the transcription factor Nrf2, a member of the Cap ‘N’ Collar (CNC) family, and a basic leucine zipper (bZIP). Its cytoplasmic repressor, Keap1, actively participates in the regulation [127]. Nrf2 movement is contingent on the relative strength of nuclear import and export signals. Nrf2 possesses three nuclear localization and two nuclear export sequences. MALAT1 acts as an antagonist in Nrf2 activation [128].

Under high expression of Sp1 and MALAT1, Keap1 performs the function of a binder by binding to Nrf2, which is subjected to ubiquitin proteinase enzymes in the cytoplasm. Keap1, acting as an oxidative stress sensor through its cysteine residues, releases Nrf2 when thiol groups undergo oxidation [129,130]. In addition to cysteine changes, both Keap1 and Nrf2 are substrates for the Cullin3/Rbx1 ubiquitin ligase adaptor protein [131]. Keap1 anchors Nrf2 by constantly directing the ubiquitin ligase to degrade it.

The oxidation liberation allows Nrf2 to enter the nucleus and initiate the transcription of genes featuring antioxidant response elements (AREs), encompassing both mitochondrial and nonmitochondrial antioxidant proteins [130,132]. Nrf2 is more likely to remain in the nucleus after heterodimerizing with the Maf protein. This Nrf2-Maf heterodimer, as a master regulator in stress situations, activates genes with AREs or electrophile response elements (EpREs). Subsequently, Keap1 then enters the nucleus along with Nrf2, which is subsequently broken down by ubiquitin enzymes, resulting in a drop in Nrf2 levels and the end of activation. Furthermore, Nrf2 is frequently reported to be downregulated or disrupted in a broad spectrum of disorders, including inflammation-related conditions, CVD, aging, and cancer [133].

## 9. ROS Accumulation by Targeting Nrf2

In 2018, Chen et al. delved into the mechanisms underlying insulin tolerance and reactive oxygen species (ROS) generation in MALAT1 null mice. Their research showed that MALAT1 blocks Nrf2 from activating, resulting in increased antioxidant machinery and lowering the production of ROS in MALAT1 null mice [128]. Nrf2, a master transcription factor, governs redox equilibrium and changes in inflammation in cells by controlling the expression of antioxidant genes in response to oxidative stress [134]. Amodio et al. (2018) discovered that MALAT1 regulates Nrf2, suggesting a significant role for MALAT1 in Nrf2 regulation [135]. Inhibiting MALAT1 could potentially protect against oxidative damage in diabetic retinopathy by influencing antioxidant defense through the Keap1-Nrf2 pathway [115]. In response to ROS accumulation, Nrf2 has the ability to enhance the production and activity of antioxidant enzymes like heme oxygenase 1 (HO-1), superoxide dismutase 2 (SOD2), and NAD(P)H quinone oxidoreductase 1 (NQO1) by going inside the nucleus, which breaks down heme and scavenges ROS under physiological conditions [136,137].

With a higher expression of MALAT1 and Keap1, Nrf2 is unable to go inside the nucleus, resulting in a reduction of antioxidant enzymes and ROS generation. Moreover, the potential depression of antioxidant gene expressions mediated by Nrf2/ARE may also result from MALAT1 ablation, given its interaction with Nrf2 in the nucleus, where it acts as a transrepressor for Nrf2 [128]. Furthermore, MALAT1 has been implicated in inducing inflammasome activation through distinct mechanisms in various diseases [138,139,140]. Yu et al. observed MALAT1’s ability to increase NLRP3 inflammasome expression in the damaged heart [138]. ROS sequestration inhibits NLRP3 inflammasome activation, attenuating hypertension-related inflammation [16]. The NLRP3 complex, once activated, is responsible for processing and secreting pro-inflammatory cytokines, leading to pyroptotic cell death and abnormal activity associated with inflammation, which ends with hypertension and multiple diseases. The study also showed that increased MALAT1 expression was followed by the increased expression of inflammation-regulating genes, such as *SAA3*, *TNF*, and *IL 6* [141]. This suggests that the therapeutic modification of MALAT1 can interfere with inflammatory processes associated with nephropathy.

Together, these findings collectively suggest that MALAT1 promotes inflammation by interacting with Keap1, thereby epigenetically suppressing Nrf2 and resulting in elevated levels of ROS and further consequences.

## 10. MALAT1 in Pulmonary Hypertension

The hemodynamic criteria defining pulmonary hypertension (PH) involve a resting mean pulmonary arterial pressure exceeding 20 mm Hg [142]. PH is characterised by significant remodeling of the distal pulmonary vasculature, marked by abnormal fibroblast growth, endothelial cell death, smooth-muscle cell hyperplasia, inflammatory cell recruitment, and collagen disruption [143]. ECs exhibit an upregulation of the lncRNA MALAT1 in response to hypoxia. When small interfering RNA (siRNA) targets MALAT1, it effectively diminishes its expression, leading to the inhibition of endothelial cell proliferation and potentially impeding vascularization [69].

In lungs from PH rats, a research investigation identified a decrease in miR-503 expression [144]. MiR-503 targets Toll-like receptor 4 (TLR4). Plasma levels of MALAT1 are significantly elevated in patients with pulmonary arterial hypertension (PAH). MALAT1, by acting as a sponge for miR-503, increases *TLR4* expression. This process inhibits apoptosis and promotes proliferation and migration in PASMCs through the miR-503-TLR4 signaling axis. MALAT1 emerges as a potential biomarker for PAH identification [95]. In hypoxic PH, the transcription factor KLF5 governs vascular remodeling by activating hypoxia-inducible factor 1α (HIF1α) [145]. MALAT1, in turn, regulates *KLF5* expression, targeted by miR-124-3p.1. Down-regulated MALAT1 in PASMCs promotes miR-124-3p.1 expression, leading to the inhibition of *KLF5* expression and subsequent reductions in cell proliferation and migration [91]. Furthermore, HIF1α increases MALAT1 expression in hypoxia-treated HPASMCs.

## 11. MALAT1 in Cardiovascular Disease

The lncRNA MALAT1 has emerged as a biomarker for CVD, and recent research suggests its involvement in the development of atherosclerosis [35,53,146,147]. Silencing MALAT1 has been shown to reduce endothelial inflammation induced by oxidized low-density lipoprotein (LDL) and protect the endothelium from oxidative stress, both critical factors in atherosclerosis development [146]. Another study reveals that MALAT1 regulates ox-LDL-associated macrophages, influencing lipid metabolism and linking to inflammatory reactions, contributing to the pathological progression of atherosclerosis [147]. Furthermore, MALAT1 interacts with the harmful mediator miR-125b, which regulates heart inflammation and promotes acute myocardial infarction [148,149,150]. For instance, MALAT1 levels were found to be elevated in coronary heart disease (CHD) patients compared to controls, while miR-125b was downregulated. These findings suggest that MALAT1 is associated with an increased risk of CHD, showing connections with altered lipid profiles, inflammation, and coronary artery stenosis. These interactions with miR-125b contribute to elevated disease severity and diminished prognosis [151]. Additionally, MALAT1 overexpression in cardiomyocytes is linked to its role in modulating electrical activity via miR-200c signaling. MALAT1 deletion may result in increased transient outward potassium current and Kv4.2/Kv4.3 channel protein expression, potentially influencing the development of arrhythmias [152].

## 12. MALAT1: Therapeutic Implications

**Figure 2:**
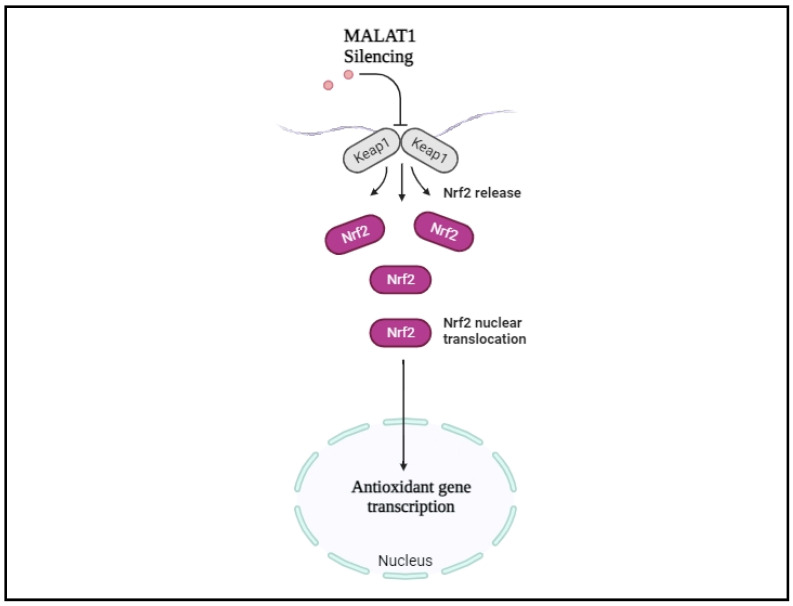
Inhibition of MALAT1 as a promising therapeutic target in salt-sensitive hypertension. (MALAT1 inhibition leads to the prevention of cardiovascular complications and inflammation following cellular salt stress stimuli. Various cellular stressors, such as salt stress and other metabolic stress by upregulating MALAT1, are involved in inflammatory pathways and antioxidant gene transcription pathways. MALAT1 knockdown induces Nrf2 to detach from its inhibiter Keap1 and move to nucleus. Nuclear Nrf2 can increase the expression of antioxidant genes such as, HO-1 and SOD-2, and the subsequent effect in salt-sensitive mechanism. The inhibition of MALAT1 would be an ideal strategy for treating cardiovascular complications such as salt-sensitive hypertension. Thus, by inhibiting MALAT1, we will be able to preserve the health and homeostasis of cardiovascular tissues).

While the presence of ncRNAs in the bloodstream holds promise as a biomarker, the utilization of treatments involving lncRNAs is still in its early stages. Despite the association of aberrant lncRNA expression or mutations with various disorders, including hypertension, ongoing research into unraveling their molecular functions provides a positive outlook. A prominent lncRNA, MALAT1, is regulated by factors such as oxidative/metabolic stress, ischemia/hypoxia, salt, and infections. MALAT1 may influence the expression and/or activities of markers related to inflammation, cell death, pyroptosis (IL-6, TNF-α, NLRP3, and caspase-1), and fibrosis (TGF-β1, FN) in cardiorenal tissues [138,139,148,153,154]. In the context of salt-sensitive hypertension disorders, MALAT1 inhibition emerges as a promising target among the lncRNA family (Figure 2).

A critical consideration in developing therapeutic targets is ensuring that deleting or downregulating these targets does not disrupt normal physiology and homeostasis. Research indicates that downregulating MALAT1 does not disrupt normal physiology and can effectively treat disease complications after metabolic stress in the post-developmental stage [155]. The next challenge in treatment development is determining the optimal delivery medium/carrier for siRNA or antisense oligonucleotides (ASO) targeting MALAT1. Not as much exploration has been performed on RNA-based therapeutic approaches for salt-sensitive hypertension disorders despite their increasing prominence over the past five years, especially in cancer and other diseases. Additionally, RNA-based therapeutic research (clinical trial) is ongoing [156] or in progress [157], including in the Food and Drug Administration (FDA)- and European Commission-approved siRNA-based medication for polyneuropathy treatment [158,159]. Numerous advancements suggest the potential for RNA-based treatments targeting MALAT1 for cardiorenal issues [155]. This can be achieved by either directly targeting MALAT1 with siRNA- or ASO-mediated knockdown or indirectly targeting it by elevating miRNAs that potentially downregulate MALAT1 expression [155].

## 13. Conclusions

In conclusion, the findings of our research suggest that upregulated MALAT1 may have played a significant role in the regulation of salt-sensitive hypertension. Downregulating MALAT1 and targeted Nrf2/antioxidant gene transcription have demonstrated improvements in endothelial functioning, reduced mRNA expression of associated variables (including those related to inflammation, endothelial function, and oxidative stress), and a decrease in apoptosis of ECs. This novel approach offers a potential avenue for treating salt-sensitive hypertension. However, further experimental research is essential to elucidate the precise roles of MALAT1 and Nrf2/antioxidant gene transcription in the development of salt-sensitive hypertension.

## Figures and Tables

**Table 1:** List of the potential target genes of MALAT1 with associated miRNA.

Gene Targets	miRNAs	Reference
*TLR4* (Toll-Like Receptor 4)	miR-503	[95]
	miR-182-5p	[96]
* KLF5 * (Kruppel-Like Factor 5)	miR-124-3p.1	[91]
* HK2 * (Hexokinase 2)	miR-145-5p	[51]
* NR3C2 * (Nuclear Receptor Subfamily 3 Group C Member 2)	miR-124-3p miR-135a-5p	[97]
* EDNRB * (Endothelin B receptor)	miR-150-5p	[50]
* NRXN1 * (Neurexin 1)	miR-141-3p/200a-3p	[98]
I*GF1R* (Insulin-Like Growth Factor 1 Receptor)	miR-133a-3p	[99]
*BECN1* (Beclin1)	miR-30a	[100]
*ZEB2* (Zinc Finger E-Box Binding Homeobox 2)	miR-200c	[101]
*AQP 4* (Aquaporin 4)	miR-145	[102]
*COX-2* (Cyclooxygenase 2)	miR-211-5p	[103]
*SIRT1* (Sirtuin 1)	miR-142-3p/Acts as ceRNA for miR-142-3p	[104]
PDE4D (Phosphodiesterase 4D)	miR-375	[105]
*HMGB1* (High Mobility Group Box 1)	miR-181c-5p/Acts as ceRNA for miR-181c-5p	[106]
*HMGA1* (High Mobility Group AT-Hook 1)	miR-195a-5p/Sponging miR-195a-5p to upregulate HMGA1	[107]
*PDE4D* (Phosphodiesterase 4D)	miR-375/(54)	[105]
* SRF * (Serum response factor)	miR-133/Acts as ceRNA for miR-133	[108]
* EZH2 * (Enhancer of zeste homolog 2)	miR-205	[109]

## Data Availability

All data are contained within the manuscript.
